# Site-specific growth and density control of carbon nanotubes by direct deposition of catalytic nanoparticles generated by spark discharge

**DOI:** 10.1186/1556-276X-8-409

**Published:** 2013-10-04

**Authors:** Hyungjoo Na, Jae Hong Park, Jungho Hwang, Jongbaeg Kim

**Affiliations:** 1 School of Mechanical Engineering, Yonsei University, 50 Yonsei-ro, Seodaemun-gu, Seoul 120-749, Republic of Korea

**Keywords:** Carbon nanotubes, Thermophoresis, Patterning, Spark discharge, Chemical vapor deposition

## Abstract

Catalytic iron nanoparticles generated by spark discharge were used to site-selectively grow carbon nanotubes (CNTs) and control their density. The generated aerosol nanoparticles were deposited on a cooled substrate by thermophoresis. The shadow mask on top of the cooled substrate enabled patterning of the catalytic nanoparticles and, thereby, patterning of CNTs synthesized by chemical vapor deposition. The density of CNTs could be controlled by varying the catalytic nanoparticle deposition time. It was also demonstrated that the density could be adjusted by changing the gap between the shadow mask and the substrate, taking advantage of the blurring effect of the deposited nanoparticles, for an identical deposition time. As all the processing steps for the patterned growth and density control of CNTs can be performed under dry conditions, we also demonstrated the integration of CNTs on fully processed, movable silicon microelectromechanical system (MEMS) structures.

## Background

Carbon nanotubes (CNTs) have attracted an enormous amount of attention from many researchers, who have found numerous device applications [1-3] taking advantage of their unique properties. Integrating CNTs into devices inevitably requires control of their location and/or density [4,5]. Controlling the synthetic location has been achieved mainly by depositing the metal catalysts in a controlled and patterned way for the following chemical vapor deposition (CVD) process.

Typically, patterning catalytic metals has been achieved using the lift-off technique, which consists of a conventional photolithography process and thin film deposition [6]. Alternative patterning methods such as soft lithography [7] or depositing catalytic thin films through shadow masks [8] have also been introduced. In these methods, however, either the catalytic film deposition requires a high-vacuum system [6,8] or the number of process repetitions is limited by the low durability of the stamp [7]. Although electroplating or electroless plating techniques [9-11] can be used to grow CNTs site-selectively and to control the density of the CNTs, these wet process approaches are not suitable for fully processed, movable silicon microelectromechanical system (MEMS) structures.

In this study, we used the spark discharge method to generate catalytic aerosol nanoparticles for CNT synthesis and patterned the particle-deposited area using a shadow mask and the thermophoresis effect [12,13]. With the patterned nanoparticles, site-specific growth of CNTs was demonstrated. Moreover, the blurring effect caused by adjusting the gap distance between the shadow mask and the substrate allowed us to deposit the catalytic nanoparticles with adjustable and location-specific densities so that the density of synthesized CNTs could be controlled differently on a single substrate. The spark discharge technique is performed using simple equipment without any high-vacuum system, and it generates and deposits the catalytic nanoparticles under dry conditions and atmospheric pressure. In addition, the shadow mask can be used repeatedly without clogging or chemical damage, and all the fabrication steps including thermophoresis are scalable to wafer scale. Furthermore, it is possible to integrate CNTs directly onto microstructures with high aspect ratios utilizing the shadow masking technique, which is difficult with conventional photolithographic patterning of a catalytic layer.

The exemplary applications of the suggested process could be field emission devices and gas sensors. Many of field emitters adopt CNTs for their electron emission tips, and the density of CNTs in this case is directly related to the current density of the device. Hence, it is important to adjust the density of CNTs which enables the device to get the desired field emission performance [14,15]. So the suggested process which can control the density of CNTs may be used as in this application. In addition, a gas sensor is usually fabricated as resistor type where the target gas is absorbed onto CNTs and changes the resistance of CNTs connecting the electrodes. Because the sensitivity of the sensor and the density of CNTs are closely related, it is needed to adjust the density of CNTs [16]; thus, this process could be also used to fabricate the gas sensor with enhanced sensitivity.

## Methods

**Figure 1:**
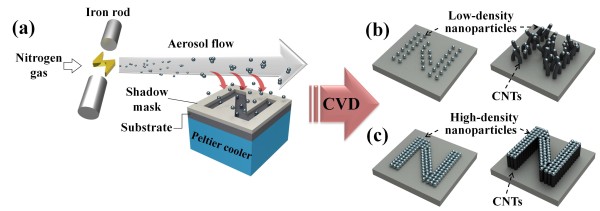
**Schematics of spark discharge process and patterned growth of CNTs with different densities. (a)** Schematic of nanoparticle generation and deposition process. Aerosol nanoparticles were generated by spark discharge and passed onto the cooled substrate sitting on the Peltier cooler. In the aerosol, small nanoparticles moved to the substrate because of the thermophoresis effect and were deposited through a hole in the patterned mask. The quantity of deposited nanoparticles is proportional to the deposition time. **(b)** A short deposition time leads to low-density CNTs. **(c)** After enough deposition time, vertically aligned CNTs grow.

Figure 1 shows schematic diagrams of the spark discharge process, selective deposition of aerosol nanoparticles, and the resultant site-specific growth of CNTs. As shown in Figure 1a, the nanoparticle generation system consists of two separated iron rods for the spark discharge and a Peltier cooler for the thermophoretic deposition of nanoparticles. When the potential difference between the isolated anode and cathode (two iron rods) was high enough, the accumulated charges were discharged through electrical breakdown in the form of a spark, vaporizing the electrodes and nucleating primary particles of a few nanometers in diameter (before agglomeration). These nanoparticles were carried by a flow of nitrogen gas and grew in size up to tens of nanometers to 100 nm by coalescence depending on the kind of metals [12,13]. Then, the aerosol nanoparticles were deposited on a silicon dioxide (SiO_2_) substrate through the patterned holes in the shadow mask because of the thermophoresis effect, in which the particles move from a high-temperature to a low-temperature area along the temperature gradient between the room-temperature aerosol nanoparticles and the bottom of the SiO_2_ substrate cooled to near 0°C by the Peltier cooler. It is known that during this thermophoretic process, smaller nanoparticles are more easily affected and moved by the temperature gradient [12,13], and thus, the majority of the nanoparticles on the patterns would be less than 100 nm in diameter. Then, the nanoparticles generated from the spark discharge were used as seed catalytic nanoparticles for CNT synthesis.

We were able to analyze the size distribution of the nanoparticles before deposition through a scanning mobility particle sizer (SMPS). The aerosol that flowed into SMPS through nitrogen at 500 sccm was analyzed for 150 s to measure the size and number of the nanoparticles, and the measurement was repeated five times to calculate the average value. Through this analysis, we were able to find the size distribution of nanoparticles in the aerosol; the diameter of the nanoparticles was distributed from 4.5 to 165.5 nm, and the mean diameter was 40.8 nm.

CNTs were synthesized by thermal CVD in a furnace. The SiO_2_ substrate was separated from the shadow mask and loaded into the quartz tube of the furnace for thermal CVD at a pressure of several millitorr. Nitrogen gas was passed through the quartz tube to prevent the oxidation of the iron catalyst and to clean the inside while the temperature was increasing up to 700°C. When the temperature stabilized, the carrier gas was replaced with a mixture of ammonia gas and acetylene gas for 10 min. In order to grow CNTs vertically, a mixture ratio of 3:1 was used, i.e., 90 sccm of ammonia gas and 30 sccm of acetylene gas [17].

## Results and discussion

**Figure 2:**
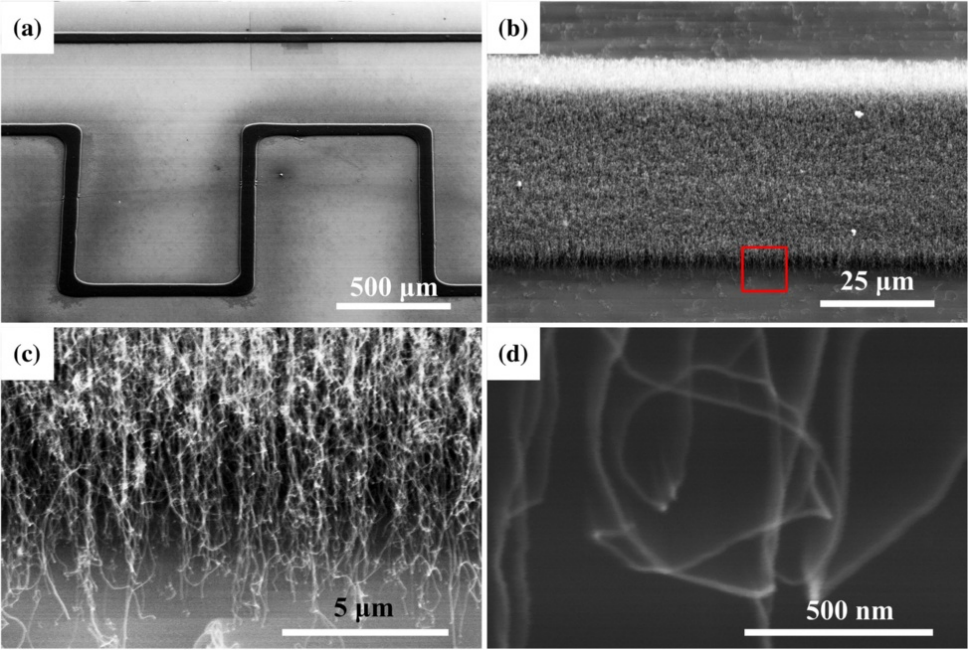
**SEM images of selectively grown CNTs. (a)** SEM image showing site-specific CNT growth. **(b)** Angled view of aligned CNTs showing the distinct edge of the pattern line. **(c)** Close-up view of the squared area in **(b)**, showing the vertically aligned CNTs grown. **(d)** High-magnification SEM image showing the individual CNTs.

Scanning electron microscope (SEM) images of a patterned CNT line are shown in Figure 2. To confirm that a clear pattern of densely grown CNTs could be formed, we deposited the catalyst for 1 h and synthesized CNTs by supplying the mixture of ammonia gas and acetylene gas for 10 min. As shown in Figure 2b,c, clearly patterned and aligned CNTs were synthesized. The 100-μm-thick stainless steel shadow mask was laser-cut to form continuous line patterns of 100 μm in width. However, the CNTs patterned through these 100-μm-wide line patterns were about 43 μm in width, as shown in Figure 2. This reduction in the line width was caused by the temperature gradient induced by the Peltier cooler, as described in previous work [12,13]. As the center of the patterns is at a lower temperature than the edge during the cooling, the majority of the nanoparticles deposited by thermophoresis will be accumulated in the narrower central region of each pattern. The average diameter of the individual CNTs shown in Figure 2d was estimated to be 30 to 50 nm.

**Figure 3:**
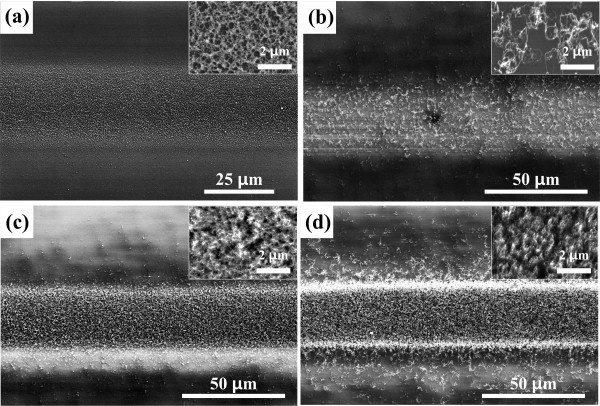
**Line patterns of CNTs by varying the catalytic nanoparticle deposition time. (a)** SEM image of the Fe nanoparticle pattern before the CVD process. The catalyst deposition time is 60 min, and the pattern width is about 30 μm. **(b)** to **(d)** SEM images showing CNTs synthesized for different catalytic nanoparticle deposition times: **(b)** 5, **(c)** 10, and **(d)** 40 min. The pattern width is about 30 μm. At least 10 min of catalyst deposition was needed to grow dense CNTs. Insets in **(a)** to **(d)** are at high magnification, and the scale bars are 2 μm.

We first varied the catalytic nanoparticle deposition time to observe its effect on the density of the grown CNTs. Figure 3a shows the nanoparticles deposited through the shadow mask for 1 h. The patterned line width is about 30 μm for a shadow mask width of 100 μm. The insets are close-up views for each panel, and the scale bar is 2 μm. Figure 3b,c,d shows the CNTs synthesized with different catalytic nanoparticle deposition times: 5, 10, and 40 min, respectively. Randomly oriented and tangled CNTs grew with a low density around the low-density catalytic nanoparticles deposited for 5 min, as shown in Figure 3b. Figure 3c,d shows the growth around the nanoparticles deposited for 10 and 40 min, respectively, where the CNTs were synthesized with a higher density and the pattern boundary was clear. The CNT line patterns had a consistent width of about 30 μm for all deposition times tested up to 40 min. From these results, we conclude that vertically aligned CNTs can grow on nanoparticles deposited for 10 min or longer. This observation matches well with the previously reported finding that the catalytic particles must have sufficient density to achieve vertical growth of CNTs [18].

As shown in Figure 3b, there were CNTs of low density with an unclear pattern when the deposition time was less than 10 min. However, with over 10 min of catalytic nanoparticle deposition time, vertically aligned CNTs were grown with high density forming a clear line pattern. Moreover, we found that the density of CNTs decreased and pattern fidelity deteriorated due to CNTs grown outside the pattern as shown in Figure 3d when the catalytic nanoparticle deposition time was over 40 min. In conventional synthesis result using Fe thin film catalyst, when the Fe thin film deposited is too thin or thick, the quality of CNTs such as density, directionality, and length becomes worse [19]. For a similar reason, it is considered that the density of CNTs is decreased by depositing an excessive amount of catalytic Fe nanoparticles through the spark discharge method. The longer deposition time may also cause an excessive blurring effect of line patterns, increasing the number of CNTs grown outside the pattern and making the pattern fidelity worse. It is concluded from this experiment that there would be an optimized deposition time for clear pattern boundaries and high density of CNTs in the proposed method, and the excessive deposition of catalytic particles resulted in blurred boundary of CNT pattern and reduced density of the CNTs grown.

**Figure 4:**
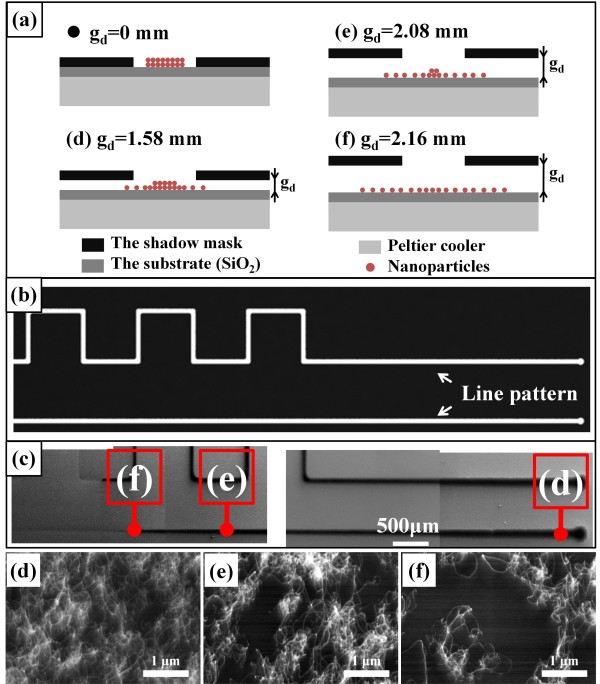
**Density-controlled growth of CNTs using the tilted mask. (a)** Schematic image showing control of the density of deposited nanoparticles using the tilted mask. The angle between the mask and the substrate is 4.76°. The **(d)** to **(f)** in **(a)** represent the distances and blurring of the deposited particles at the corresponding positions, **(d)** to **(f)** in **(c)**. The distances between the mask and the substrate at points **(d)** to **(f)** are 1.58, 2.08, and 2.16 mm, respectively. **(b)** Schematic of the shadow mask with line pattern. **(c)** Arranged SEM images to show the CNT line pattern. **(d)** to **(f)** High-magnification images of each point in **(c)** clearly showing the density difference.

The gap distance between the substrate and the shadow mask also influenced the density of the deposited catalyst. The nanoparticles spread out when they pass through the patterns of the shadow mask, and the larger the gap is, the more spreading is observed, resulting in a reduction in the density of the particles on the deposited region. To utilize this blurring effect to adjust the density of the grown CNTs, we tilted the shadow mask such that the gap distance between the shadow mask and the substrate changed linearly, as shown in Figure 4a. For this experiment, we used a shadow mask tilted at an angle of 4.76° with respect to the substrate surface, and the gap distance varied linearly from 0 to 4 mm. Figure 4b shows the schematic of the shadow mask pattern used for the CNT line pattern of SEM images shown in Figure 4c. The stainless steel mask is the same as the one used in other experiments and has a length and width of 48 mm × 22 mm and 100 μm of thickness. The width of the laser-cut line pattern is 100 μm. Figure 4d,e,f shows the different site densities of CNTs at the positions illustrated in Figure 4c, where the heights of the shadow mask from the substrate were 1.58, 2.08, and 2.16 mm, respectively. As expected, when the distance between the shadow mask and the substrate was increased, the density of CNTs progressively decreased and the line became wider because of the blurring. The CNT line pattern looks broken when viewing the location of (f) in Figure 4c. The reason for the unclear pattern on the left side of (f) is a reduction of the density of CNTs due to an increase of the blurring effect caused by the receded gap distance between the substrate and the shadow mask. Using this approach, we could gradually vary the density of catalytic nanoparticles and thus gradually change the density of CNTs on a single substrate with a single run of the synthetic process.

**Figure 5:**
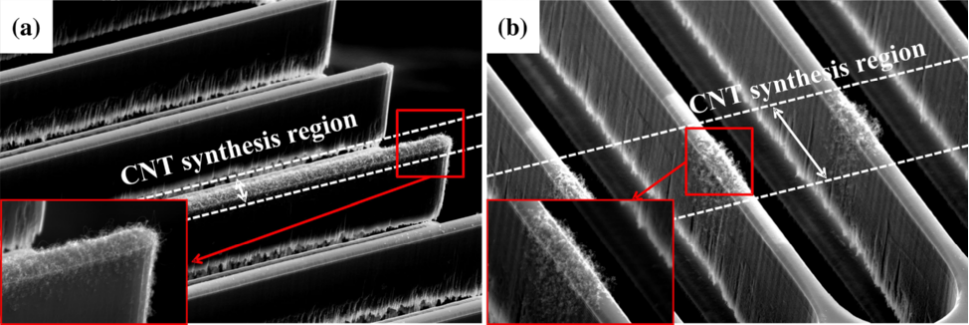
**CNTs on MEMS structure.** The catalyst was deposited selectively on a comb structure parallel and perpendicular to the shadow mask. SEM images show CNTs grown with the **(a)** parallel and **(b)** perpendicular alignment between the shadow mask and the comb structure. The insets are the close-up views of the CNTs in red squares.

We also demonstrated that this method could be applied to the growth of CNTs on a designated area of released micromechanical structures. Typically, released and movable microstructures fail by stiction when the structures are exposed to a liquid, and thus, this demonstration was only possible because no wet process was involved in our proposed method. Figure 5 shows the CNTs synthesized on the released comb structures formed on a device layer of a silicon-on-insulator (SOI) wafer. In Figure 5a, the CNTs were grown on a desired comb only, while the CNTs were grown in a band across multiple combs in Figure 5b. The insets in each figure are the close-up views of CNTs in red squares.

## Conclusions

In conclusion, we demonstrated for the first time that the nanoparticles generated using the spark discharge method can be used successfully as catalysts for the growth of CNTs. The nanoparticles were transferred onto the desired area on a substrate by thermophoresis and were patterned using a shadow mask to realize patterned growth of CNTs. The nanoparticle deposition time determines the final density of the grown CNTs, and vertically aligned growth of CNTs was achieved after 10 min of nanoparticle deposition in our experiment. An alternative approach to changing the density of CNTs was to change the gap between the shadow mask and the substrate, and a patterned line of CNTs with gradually varying density along the line could be formed by tilting the shadow mask. The proposed all-dry process could also be applied to completely fabricated micromechanical structures, as demonstrated by site-specifically growing the CNTs on the released high-aspect-ratio microstructures.

## Abbreviations

CNTs: Carbon nanotubes; CVD: Chemical vapor deposition; MEMS: Microelectromechanical systems; SEM: Scanning electron microscopy; SiO2: Silicon dioxide; SOI: Silicon-on-insulator.

## Competing interests

The authors declare that they have no competing interests.

## Authors' contributions

HN carried out the synthesis of CNTs and drafted the paper. JHP and JH worked on the spark discharge experiment. JK supervised all the works and revised the manuscript. All authors read and approved the final manuscript.

## References

[B1] Frank S Poncharal P Wang ZL de Heer WA Carbon nanotube quantum resistors Science 1998 8 1744 10.1126/science.280.5370.1744 9624050

[B2] Tans SJ Verschueren ARM Dekker C Room-temperature transistor based on a single carbon nanotube Nature 1998 8 49 10.1038/29954

[B3] Kong J Franklin NR Zhou C Chapline MG Peng S Cho K Dai H Nanotube molecular wires as chemical sensors Science 2000 8 622 10.1126/science.287.5453.622 10649989

[B4] Murakami H Hirakawa M Tanaka C Yamakawa H Field emission from well-aligned, patterned, carbon nanotube emitters Appl Phys Lett 2000 8 1776 10.1063/1.126164

[B5] Siegal MP Overmyer DL Kaatz FH Controlling the site density of multiwall carbon nanotubes via growth conditions Appl Phys Lett 2004 8 5156 10.1063/1.1765741

[B6] Jeong G Olofsson N Falk LKL Campbell EEB Effect of catalyst pattern geometry on the growth of vertically aligned carbon nanotube arrays Carbon 2009 8 696 10.1016/j.carbon.2008.11.003

[B7] Kind H Bonard J Patterned films of nanotubes using microcontact printing of catalysts Adv Mater 1999 8 1285 10.1002/(SICI)1521-4095(199910)11:15<1285::AID-ADMA1285>3.0.CO;2-J

[B8] Fan S Chapline MG Franklin NR Tombler TW Cassell AM Dai H Self-oriented regular arrays of carbon nanotubes and their field emission properties Science 1999 8 512 10.1126/science.283.5401.512 9915692

[B9] Hwang SK Jeong SH Lee KH Packing density control of carbon nanotube emitters in an anodic aluminum oxide nano-template on a Si wafer Diam Relat Mater 2006 8 1501 10.1016/j.diamond.2005.11.023

[B10] Tu Y Huang ZP Wang DZ Wen JG Ren ZF Growth of aligned carbon nanotubes with controlled site density Appl Phys Lett 2002 8 4018 10.1063/1.1482790

[B11] Chao CW Wu YS Hu GR Feng MS Selective growth of carbon nanotubes on prepatterned amorphous silicon thin films by electroless plating Ni J Electrochem Soc 2003 8 C631 10.1149/1.1596953

[B12] Byeon JH Yoon KY Jung YK Hwang J Thermophoretic deposition of palladium aerosol nanoparticles for electroless micropatterning of copper Electrochem Commun 2008 8 1272 10.1016/j.elecom.2008.06.025

[B13] Byeon JH Park JH Yoon KY Jung YK Hwang J Site-selective catalytic surface activation via aerosol nanoparticles for use in metal micropatterning Langmuir 2008 8 5949 10.1021/la8005019 18459805

[B14] Bonard J-M Weiss N Kind H Stöckli T Forró L Kern K Châtelain A Tuning the field emission properties of patterned carbon nanotube films Adv Mater 2001 8 184

[B15] Nilsson L Groening O Emmenegger C Kuettel O Schaller E Schlapbach L Kind H Bonard J-M Kern K Scanning field emission from patterned carbon nanotube films Appl Phys Lett 2071 8 76

[B16] Suehiro J Zhou G Imakiire H Ding W Hara M Controlled fabrication of carbon nanotube NO_2_ gas sensor using dielectrophoretic impedance measurement Sensor Actuat B-chem 2005 8 398 10.1016/j.snb.2004.09.048

[B17] Liu J Webster S Carroll DL Temperature and flow rate of NH_3_ effects on nitrogen content and doping environments of carbon nanotubes grown by injection CVD method J Phys Chem B 2005 8 15769 10.1021/jp050123b 16853001

[B18] Murakami Y Chiashi S Miyauchi Y Hu M Ogura M Okubo T Maruyama S Growth of vertically aligned single-walled carbon nanotube films on quartz substrates and their optical anisotropy Chem Phys Lett 2004 8 298 10.1016/j.cplett.2003.12.095

[B19] Wang Y Luo Z Li B Ho PS Yao Z Shi L Bryan EN Nemanich RJ Comparison study of catalyst nanoparticle formation and carbon nanotube growth: support effect J Appl Phys 2007 8 124310 10.1063/1.2749412

